# The Tail Associated Protein of *Acinetobacter baumannii* Phage ΦAB6 Is the Host Specificity Determinant Possessing Exopolysaccharide Depolymerase Activity

**DOI:** 10.1371/journal.pone.0153361

**Published:** 2016-04-14

**Authors:** Meng-Jiun Lai, Kai-Chih Chang, Shiuan-Wen Huang, Cheng-Hung Luo, Pei-Yu Chiou, Chao-Chuan Wu, Nien-Tsung Lin

**Affiliations:** 1 Department of Laboratory Medicine and Biotechnology, Tzu Chi University, Hualien, Taiwan; 2 Master Program in Microbiology and Immunology, School of Medicine, Tzu Chi University, Hualien, Taiwan; 3 Institute of Medical Sciences, Tzu Chi University, Hualien, Taiwan; 4 Department of Surgery, Buddhist Tzu Chi General Hospital, Taipei, Taiwan; Centro Nacional de Biotecnologia - CSIC / CIF Q2818002D, SPAIN

## Abstract

*Acinetobacter baumannii* is a non-fermenting, gram-negative bacterium. In recent years, the frequency of *A*. *baumannii* infections has continued to increase, and multidrug-resistant strains are emerging in hospitalized patients. Therefore, as therapeutic options become limited, the potential of phages as natural antimicrobial agents to control infections is worth reconsidering. In our previous study, we isolated ten virulent double-stranded DNA *A*. *baumannii* phages, ϕAB1–9 and ϕAB11, and found that each has a narrow host range. Many reports indicate that receptor-binding protein of phage mediates host recognition; however, understanding of the specific interactions between *A*. *baumannii* and phages remains very limited. In this study, host determinants of *A*. *baumannii* phages were investigated. Sequence comparison of ϕAB6 and ϕAB1 revealed high degrees of conservation among their genes except the tail fiber protein (ORF41 in ϕAB1 and ORF40 in ϕAB6). Furthermore, we found that ORF40^ϕAB6^ has polysaccharide depolymerase activity capable of hydrolyzing the *A*. *baumannii* exopolysaccharide and is a component of the phage tail apparatus determining host specificity. Thus, the lytic phages and their associated depolymerase not only have potential as alternative therapeutic agents for treating *A*. *baumannii* infections but also provide useful and highly specific tools for studying host strain exopolysaccharides and producing glycoconjugate vaccines.

## Introduction

*Acinetobacter baumannii* is a non-fermenting, gram-negative bacterium. According to the 2014 report by the Center for Disease Control, Taiwan, *A*. *baumannii* is an emerging pathogen ranked first among gram-negative bacteria that can cause outbreaks of nosocomial infections such as bloodstream infections, endocarditis, ventilator-acquired pneumonia, meningitis, urinary tract infections, and wound infections with rising antibiotic resistance rates [1]. Therefore, as therapeutic options become limited, the search for novel agents becomes a priority. To fight this bacterium, one of the promising alternative approaches to conventional antibiotics treatment is phage therapy. Bacteriophages (phages) are non-cellular microorganisms that infect and lyse specific bacteria. Phages and their products are receiving renewed attention as bioagents for the treatment or prophylaxis of bacterial infectious diseases. However, the high host specificity of phages narrows the potential application of phage therapy. Therefore, an understanding of phage specificity is needed to predict the success and consequences of phage therapy.

The first step of phage infection is adsorption of the phage virion to a susceptible host cell, which is determined by the specific interaction between phage receptor-binding protein (RBP) and a specific receptor on the surface of the host cell [2–4]. To overcome the host specificity barriers, it is vital to understand the appendages of phages used to recognize the receptors on the host cell surface. The detailed recognition mechanisms vary among phages [5]. Most of the information on RBPs and phage-host interactions comes from studies carried out on coliphages and lactic acid phages. These RBPs have been identified as the central tail fibers or protruding baseplate proteins and have been well studied. For example, the major host range determinant in T-even phages is the distal end of their long tail fibers, which recognizes receptors on the surface of the *Escherichia coli* host [6]. Wang et al. [7] demonstrated that only the C-terminal 249 amino acids of the tail fiber protein in λ phage could bind with the LamB receptor and are thus involved in host specificity. In contrast, T5 and BF23 phages use the tail shaft, which is located above the straight tail fiber, as an RBP [8,9], and the RBPs from *Lactococcal* phages TP901-1 and Tuc2009 are lower baseplate proteins [10].

Unfortunately, phage-host interactions remain poorly understood in *A*. *baumannii*, even as increasing numbers of novel phages are reported. In our previous study, we isolated ten virulent double-stranded DNA bacteriophages, ϕAB1–9 and ϕAB11, and found that each has a narrow host range [11], indicating that the RBPs vary. In addition, we previously published the complete genome of the first *A*. *baumannii* lytic phage ϕAB1 [12]. Phage ϕAB1 possesses an isometric head of 60 nm, a short tail of 9×11 nm, and a linear double-stranded DNA genome containing 41,526 bp with a GC content of 39%. We identified 46 open reading frames (ORFs), including a putative tail fiber protein assigned as ORF41. Moreover, the 150 N-terminal residues of ORF41 were similar to the N-terminal regions of gp49 from LKA1 and gp38 from ϕKMV, a conserved region found among tail fibers of the T7-like group [13] in a BLASTp analysis. Ceyssen et al. [13] reported that the C-terminal regions of the tail fiber proteins in LKA1 are responsible for binding to the host-surface receptor. Thus, based on the bioinformatic analysis, we speculate that the gene product of *orf41* is important for ϕAB1 recognition of *A*. *baumannii*: the 150 N-terminal residues are responsible for attaching the tail fibers to the phage virion, and the C-terminal region is responsible for specific binding to the host-surface receptor.

In this study, we sequenced the genome of phage ϕAB6, which belongs to the same Autographivirinae subfamily as ϕAB1 but exhibits a different host range. Analysis of the similarities and differences of these phages will provide a useful model for identifying and studying the phage genetic determinant (RBP) for host specificity.

## Materials and Methods

### Bacterial strains and phages

*A*. *baumannii* M68316 and 54149 were used as the propagation and the indicator host of ϕAB1 and ϕAB6, respectively. Phage sensitivity was tested by spot tests, which were performed by dropping 5 μL phage lysate (ca. 1×10^9^ PFU/mL) onto a lawn of *A*. *baumannii* cells in the top agar. Clearing zone formation indicated that the cells were phage sensitive.

### Plaque and adsorption assay

Phage titers were determined by plaque assay on host strain M68316 or 54149 in an LB agar plate, and adsorption assays were performed as previously described [11]. Briefly, *A*. *baumannii* cells (0.6 U of OD_600_) were infected with phages at an MOI of 0.0001, and samples (100 μL) were collected after 5-min incubation at 37°C with shaking, diluted in 0.9 mL cold LB, and centrifuged (12,000 ×*g*, 5 min). The supernatants were titrated by plaque assay. Percent adsorption of the phage was calculated as [(initial titer − residual titer in the supernatant)/initial titer] × 100%. Error bars represent the standard deviation of the three independent experiments.

### Protein expression and purification

A fragment of *orf40*^ϕAB6^, encoding a putative polysaccharide depolymerase, was amplified from purified phage ϕAB6 by PCR using the primers AB6TFF1 (5'-GGATCCATGAATATACTACGCTCATTTA-3') and AB6TFR1 (5'-GTCGACTTAACTCGTTGCTGTAAATG-3'). Underlined nucleotides indicate recognition sequences for *BamH*I and *Sal*I. The PCR fragment was excised by *BamH*I and *Sal*I inserted into the pET-30a expression vector (Novagen, Madison, WI, USA). The resulting pET-orf40^ϕAB6^ plasmid was transformed into *E*. *coli* BL21(DE3) cells. The recombinant His-tagged ORF40 protein was expressed under 0.1 mM IPTG induction at 20°C overnight then purified from the soluble fraction using a Ni-NTA column (Qiagen, Valencia, CA) according to the manufacturer’s instructions and analyzed by SDS-PAGE.

### Polyclonal antibody preparation

Polyclonal antibodies were raised in rabbits by Protech Technology Enterprise Co., Ltd. (Taipei, Taiwan) using its standard protocol. Initial immunizations of ORF40^ϕAB6^ were complemented with Freund's complete adjuvant with five subsequent booster injections of protein. Protein concentrations in the initial immunization and booster injections were 150–200 μg/mL. The final serum samples were acquired 11 weeks after the initial immunizations.

### Determination of the polysaccharide depolymerase activity

The spot test was performed to observe polysaccharide depolymerase activity [14]. In brief, LB agar in a petri dish was overlaid with 0.7% top agar inoculated with 300 μL fresh bacterial culture. Phage or purified recombinant polysaccharide depolymerase (1 μL, 1 μg/μL) was spotted onto the double-layer plate. After overnight incubation at 37°C, plates were observed for formation of lytic or semi-clear spots.

### Immunogold detection of ORF40^ϕAB6^ in virions

For gold immunolabeling, purified phage particles were dialyzed and incubated overnight at room temperature with primary antibody, anti-ORF40^ϕAB6^, diluted 1:1000 in buffer. Next, 6-nm gold-conjugated goat anti-rabbit immunoglobulin G secondary antibody (Electron Microscopy Sciences, PA, USA) diluted 1:20 (wt/wt) with buffer was added for 2 h at room temperature. After fixation for 30 min at room temperature in phosphate-buffered saline with 0.25% glutaraldehyde, virions were negatively stained with 2% uranyl acetate and visualized by transmission electron microscopy.

### ϕAB6 genome DNA sequencing

Phage ϕAB6 DNA was sequenced using pyrosequencing (454 technology). A total of 22,736 reads were assembled to a single contig at 122-fold coverage. The location of the terminal repeats was suggested using the ϕAB1 genome (GenBank accession number HQ186308) as the reference and confirmed with primer walking of the ϕAB6 DNA. Genes were predicted using GeneMark.hmm [15] and annotated using NCBI-BLAST [16]. The genomic sequence of ϕAB6 was deposited in GenBank (accession number KT339321).

### Construction of chimeric phage ϕAB1tf6

To construct the chimeric phage ϕAB1tf6 carrying the tail fiber protein (ORF40) of ϕAB6 instead of ϕAB1 ORF41, overlapping PCR was used. A fragment containing upstream *orf41* with a unique *Ban*II restriction site was PCR amplified from ϕAB1 DNA with the primers AB1gp41F (5'-AGTACACCTAGTGGGGCTCC-3') and AB16gp42FR (5'-TAGCTTCTTGTGCTTGTTGCA-3'). The DNA fragment comprising the ϕAB6 tail fiber to the DNA maturase containing a unique *Bgl*II restriction site was amplified from ϕAB6 DNA with the primers AB16gp42FF (5'-TGCAACAAGCACAAGAAGCTA-3') and AB1gp45R (5'-GTTGTGCTTCCGATAGATCTTT-3'). The ϕAB1 *orf41* and ϕAB6 *orf40* fusion DNA fragment was amplified from the two amplicons described above using overlapping PCR with primers AB1gp41F and AB1gp45R, and the region of primers AB16gp42FR and AB16gp42FF was designed to link the ϕAB1 *orf41* and ϕAB6 *orf40*. Chimeric phage ϕAB1tf6 was generated by electroporation of the ligated left *Ban*II arm, the right *Bgl*II arm of ϕAB1, and *orf40* containing the *Ban*II-*Bgl*II DNA fragment of ϕAB6 into *A*. *baumannii* strain 54149 using a Bio-Rad Gene Pulser II system at 2.5 kV and 25 μF with the pulse controller adjusted to 200 Ω. A single plaque was subsequently isolated and amplified with *A*. *baumannii* strain 54149 to check the chimeric phage ϕAB1tf6. To confirm the recombinant ϕAB1tf6 phage carried *orf40* from ϕAB6, a set of primers (pf1: 5'-GCGTAATGGTAAGGAAC-3' and pr3: 5'-TCGCATCTACTTGAGTTT-3') annealing upstream and downstream of *orf40*, which is homologous in ϕAB1 and ϕAB6, was used.

## Results

### Phage characteristics

Phages ϕAB1 and ϕAB6 were isolated from sewage water in our laboratory, as reported previously [11]. Electron microscopy revealed that they are members of Podoviridae, both with a head diameter of approximately 60 nm and a short tail with 9-nm diameter and 11-nm length [11]. In addition, the host spectrum of each phage was different: ϕAB1 was shown to infect *A*. *baumannii* strain M68316 and ϕAB6 to infect strain 54149. In this study, adsorption assays were performed to test whether host specificity was determined by initial adsorption or some other resistance mechanisms. The results demonstrated that ϕAB1 was unable to adsorb to strain 54149, and ϕAB6 failed to bind to strain M68316 (see below in section "Construction and characterization of chimeric phage ϕAB1tf6 with altered host specificity"), suggesting that ϕAB1 and ϕAB6 may encode different RBPs that are responsible for their differential host specificity. In host range test, ϕAB1 lysed 23% (191/832) of the clinical *A*. *baumannii* isolates: in contrast, ϕAB6 was able to lyse 58% (483/832) of the isolates, indicating that ϕAB6 has a much wider host range than ϕAB1.

### Genome sequencing and ORF identification for ϕAB6

To carry out molecular characterization of the phage, we previously determined the sequence for the whole genome of ϕAB1 [12]. In this study, we determined the sequence of the ϕAB6 genome. The sequencing results revealed that the ϕAB6 genome is 40,559 bp in size; includes direct terminal repeats (DTRs) of 410 bp, as determined by a combination of shotgun and primer walking approaches and confirmed by direct sequencing with outward-directed primers, leading to termination of the sequencing reaction at the ends of the genome; and has a GC content of 39%, which is similar to that of the host *A*. *baumannii*. Analysis showed that ϕAB6 (accession number KT339321) shares 82.3% DNA sequence similarity with ϕAB1 (accession number HQ186308). Potential ORFs of ϕAB6 were identified based on gene prediction tools, tBLAST homology searches, and visual inspection of potential ORFs and their Shine-Dalgarno sequences, leading to prediction of 46 ORFs. The genome organization was largely conserved with that of ϕAB1 (Table 1), although some notable differences were observed: 1) ORF6, 17, and 19 of ϕAB1 are absent from ϕAB6, and ORF9, 12, and 18 of ϕAB6 are absent from ϕAB1; 2) the DNA polymerase gene is separated into two domains (ORF18 and ORF20) by the putative HNH-AP2 endonuclease gene (ORF19) in ϕAB1 but it remains a complete ORF encoding 766 aa in ϕAB6 (ORF19); and, most interestingly, 3) there was a low degree of identity (34%) in amino acid sequence between the predicted tail fibers of ϕAB1 (ORF41) and ϕAB6 (ORF40), suggesting that the tail fiber protein might be the determinant for host specificity.

**Table 1:** Comparison of the putative open reading frames (ORFs) of ϕAB6 with ϕAB1.

ϕAB6					ϕAB1				
ORF	Start	End	Length (aa)	Predicted function	ORF	Start	End	Length (aa)	% aa identity
1	1582	2091	169	Hypothetical protein	1	1574	2083	169	95.8
2	2093	2494	133		2	2085	2486	133	96.2
3	2481	2714	77		3	2473	2706	77	100.0
4	2789	3385	198		4	2781	3377	198	99.5
5	3645	4043	132		5	3637	4035	132	99.2
					6	4025	4132	35	
6	4122	4601	159		7	4211	4690	159	100.0
7	4603	5037	144		8	4692	5126	144	99.3
8	5048	5215	55		9	5137	5304	55	100.0
9	5202	5393	63						
10	5390	5623	77		10	5478	5696	72	52.0
11	5613	5822	69		11	5686	5904	72	57.5
12	5844	6920	148	HNH endonuclease	17				45.2
					24				39,3
13	6620	7075	151	DNA primase	12	5894	6694	266	96.0
14	7075	7392	105		13	6694	7011	105	96.2
15	7392	7628	78		14	7011	7247	78	98.7
16	7642	8940	432	DNA helicase	15	7260	8558	432	100.0
17	8943	9923	326	ATP-dependent DNA ligase	16	8555	9541	328	99.4
18	9916	9999	27						
				HNH endonuclease	17	9801	10247	148	
19	10241	12541	766	DNA polymerase	18	10244	11104	286	96.2^a^
				HNH endonuclease	19	11088	11525	145	
				DNA polymerase	20	11494	12978	494	91.3^b^
20	12550	13029	159	HNH endonuclease	21	12987	13466	159	98.1
21	13047	13937	296	Hypothetical protein	22	13484	14374	296	100.0
22	14146	15120	318	DNA exonuclease	23	14583	15539	318	98.7
23	15083	15520	145	HNH endonuclease	24	15520	15957	145	95.9
24	15517	15957	146	DNA endonuclease VII	25	15954	16394	146	98.4
25	15961	16896	311	Hypothetical protein	26	16398	17333	311	100.0
26	16896	17546	216	dNMP kinase	27	17333	17983	216	96.3
27	17555	19972	805	RNA polymerase	28	17992	20409	805	98.0
28	20075	20272	65		29	20512	20709	65	98.5
29	20262	20520	83	Structural protein	30	20706	20957	83	97.6
30	20529	22805	518	Head-tail connector protein	31	20966	22522	518	99.4
31	22094	22954	286	Scaffolding protein	32	22531	23391	286	96.5
32	22970	24001	343	Capsid protein	33	23407	24438	343	98.8
33	24036	24241	61		34	24491	24676	61	95.1
34	24253	24546	97	Hypothetical protein	35	24688	24981	97	95.9
35	24737	25297	186	Tail tubular protein A	36	25106	25732	208	98.9
36	25306	27597	763	Tail tubular protein B	37	25741	28032	763	98.7
37	27597	28268	223	Internal virion protein B	38	28032	28706	224	96.3
38	28281	31166	961	Structural protein	39	28719	31604	961	98.2
39	31176	34274	1032	Internal virion (core) protein	40	31614	34712	1032	98.6
40	34281	36380	699	Tail fiber	41	34719	37367	882	95.5^C^
41	36511	36846	111	Holin	42	37484	37813	109	100.0
42	36833	37390	185	Chitinase-like endolysin	43	37800	38357	185	96.8
43	37509	37817	102	DNA maturase A	44	38476	38784	102	99.0
44	37827	39764	645	DNA maturase B	45	38794	40731	645	99.5
45	39855	40058	67		46	40822	41025	67	95.5

^a^Identity is calculated based on the first 290 residues of ORF19 in ϕAB6 aligned to ORF18 in ϕAB1.

^b^Identity is calculated based on the C terminus (amino acids 290–766) of ORF19 in ϕAB6 aligned to ORF20 in ϕAB1.

^c^Identity is calculated based on the alignment between the first 200 residues of the two proteins.

**Fig 1:**
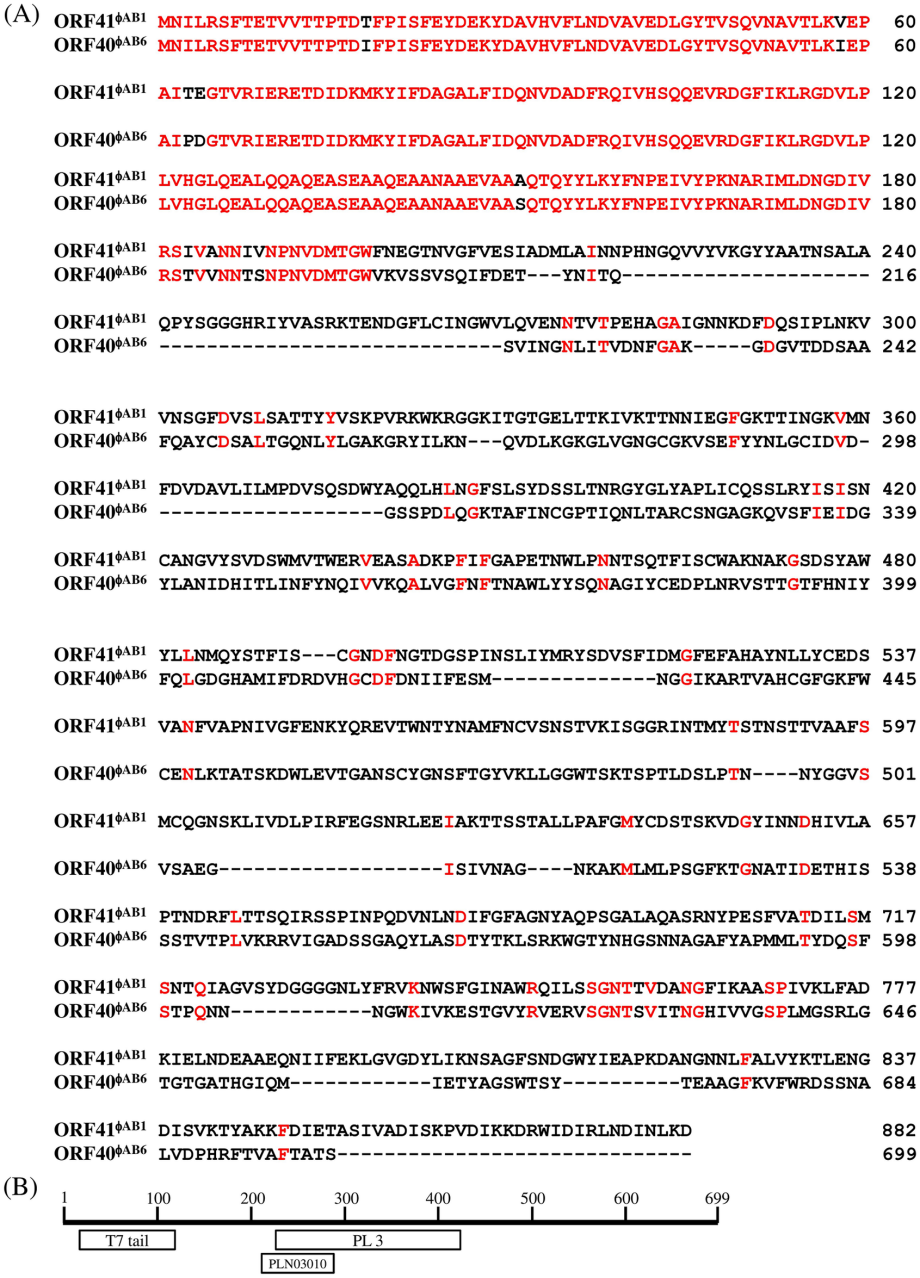
Sequence alignment of tail fibers of *A*. *baumannii* phages. (A) Amino acid sequence alignment of the phage tail fibers encoded by ϕAB1 ORF41 and ϕAB6 ORF40, respectively. (B) Predicted domain structure of ϕAB6 ORF40. The numbers on the top indicate size in amino acid residues. The domains are identified as follow: T7 tail (aa 10–112), T7 phage tail domain; PLN03010 (aa 218–274), the PLN03010 domain of polygalacturonase; and PL 3 (aa 226–419) pectate lyase 3 superfamily.

BLASTp analysis revealed that the 150-aa N-terminal domain of ORF40^ϕAB6^ is similar to the N-terminal regions of LKA1 gp49 and ϕKMV gp38, which are conserved among tail fibers of the T7-like group [13]. In addition, amino acid sequence alignment of ORF41^ϕAB1^ and ORF40^ϕAB6^ showed that their N-terminal 198 aa shared 95% identity; however, almost no similarity was observed in their C-terminal regions (Fig 1A). Further, the central domain of 194 residues (aa 226–419) of ORF40^ϕAB6^, which is not present in ORF41^ϕAB1^, has similarity to the pectate lyase 3 superfamily domain (Fig 1B), which is most closely related to glycosyl hydrolase. This finding suggests that this region may be able to hydrolyze the glucosidic bond of bacterial exopolysaccharides. These observations imply that the 198 N-terminal residues of ORF40^ϕAB6^ are responsible for attaching to the phage virion and that the C-terminal residues are responsible for host specificity and exopolysaccharide depolymerization, thus destroying the integrity of the host cell wall for phage entry.

**Fig 2:**
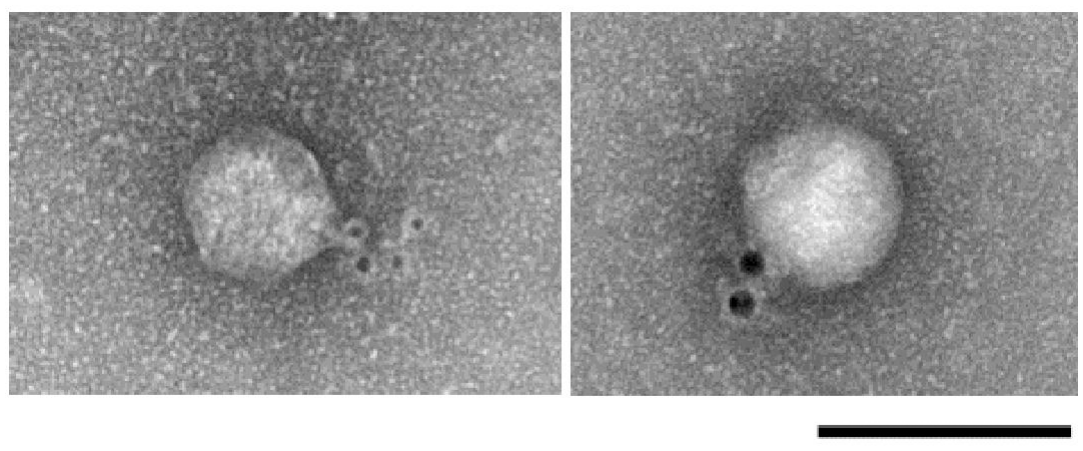
Immunogold-labeling electron microscopy of ϕAB6 phage particles. The primary antibody targeting ϕAB6 ORF40 is described in Materials and Methods. The secondary antibody was conjugated to 6-nm diameter gold particles (black dots). Scale bar, 100 nm.

To determine the location of ORF40^ϕAB6^, we performed immunogold electron microscopy analysis. As shown in Fig 2, the electron-dense gold-labeled antibodies appeared as black spots attaching to the tail of ϕAB6, demonstrating ORF40^ϕAB6^ to be the tail fiber protein.

### Analysis of the polysaccharide depolymerization activity of ORF40^ϕAB6^

**Fig 3:**
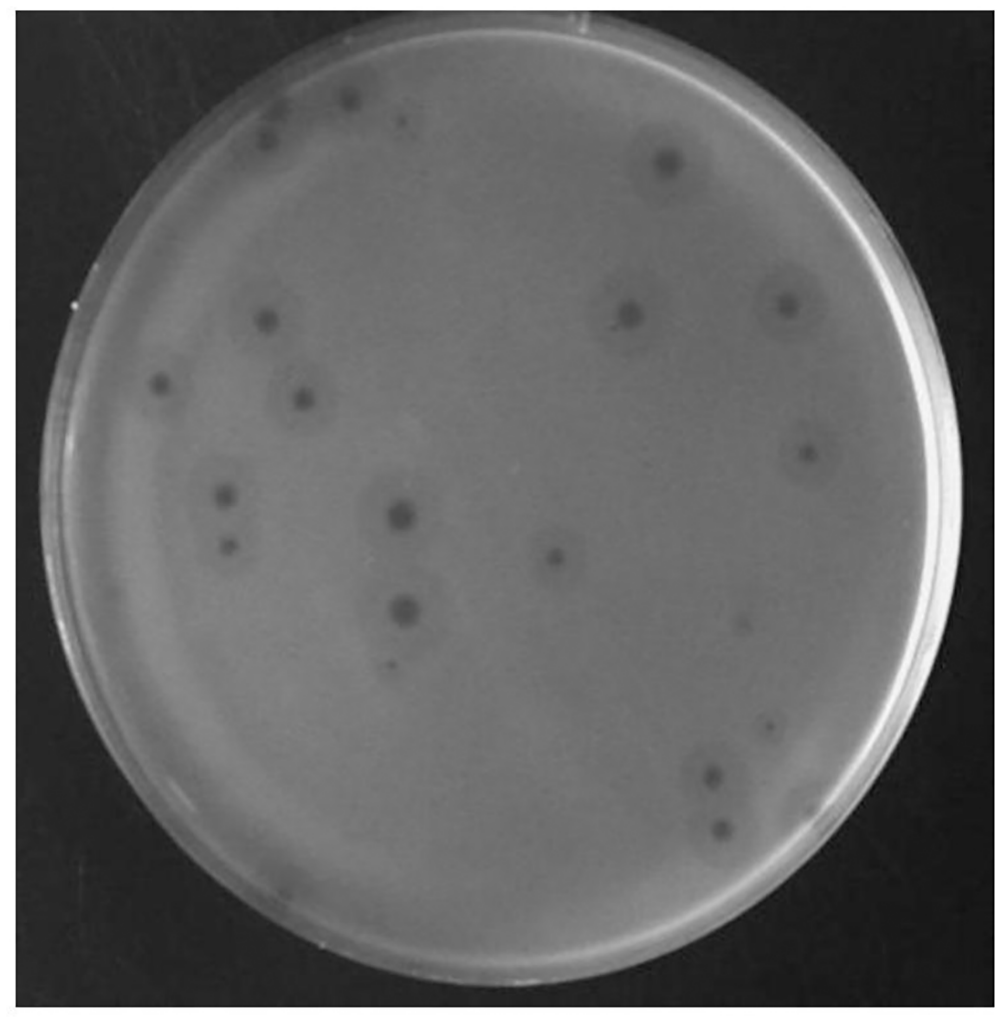
Translucent halo formation by phage ϕAB6. Clear plaques surrounded by translucent halos were observed in the plaque assay of phage ϕAB6 with *A*. *baumannii* strain 54149 as the indicator host.

**Fig 4:**
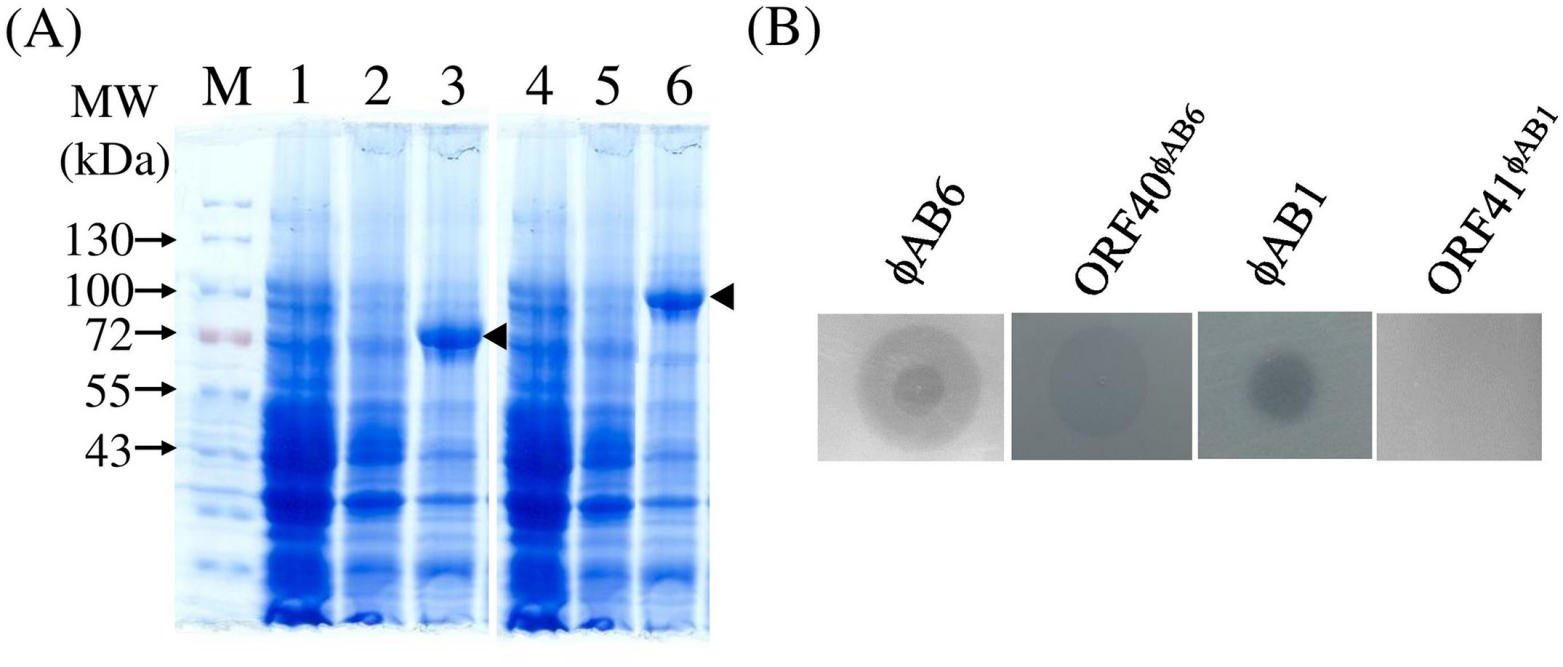
Overexpression and depolymerization activity test of ORF40^ϕAB6^ and ORF41^ϕAB1^ proteins. (A) SDS-PAGE of the recombinant proteins overexpressed. Lanes: M, protein markers; 1 and 4, BL21(DE3) harboring an empty plasmid; 2, uninduced BL21(DE3)/pET-orf40^ϕAB6^; 3, BL21(DE3)/pET-orf40^ϕAB6^ induced with 0.1 mM IPTG; 5, uninduced BL21(DE3)/pET-orf41^ϕAB1^; 6, BL21(DE3)/pET-orf40^ϕAB6^ induced with 0.1 mM IPTG. The arrowheads indicate the overexpressed recombinant proteins. (B) Enzyme activity of purified proteins was tested by spot test. Halo surrounding stands for positive activity.

The plaques manifested by ϕAB6 were surrounded by translucent halos (Fig 3), indicating that host cell exopolysaccharides were depolymerized. This phenomenon suggested that the phage produced a depolymerase enzyme that could diffuse through the agar layer to degrade bacterial exopolysaccharides into oligosaccharide units during infection. It is known that the tail spike or tail fiber often exhibits depolymerase activity [17]. To test whether this was true for the ϕAB6 tail fiber, we cloned *orf40*^ϕAB6^ into the pET30a expression vector and purified the His-tagged ORF40^ϕAB6^ protein. As expected, recombinant protein with a size of 77 kDa was obtained (Fig 4A, lane 3). In spot tests, the purified recombinant ORF40^ϕAB6^ protein caused depolymerization of the exopolysaccharide of host strain 54149, forming translucent halos, but not that of non-host strain M68316 (Fig 4B). In parallel experiments, *orf41*^ϕAB1^ was cloned and expressed in *E*. *coli*. However, the recombinant protein ORF41^ϕAB1^ (97 kDa; Fig 4A, lane 6) did not exhibit depolymerase activity (Fig 4B).

### Construction and characterization of chimeric phage ϕAB1tf6 with altered host specificity

**Fig 5:**
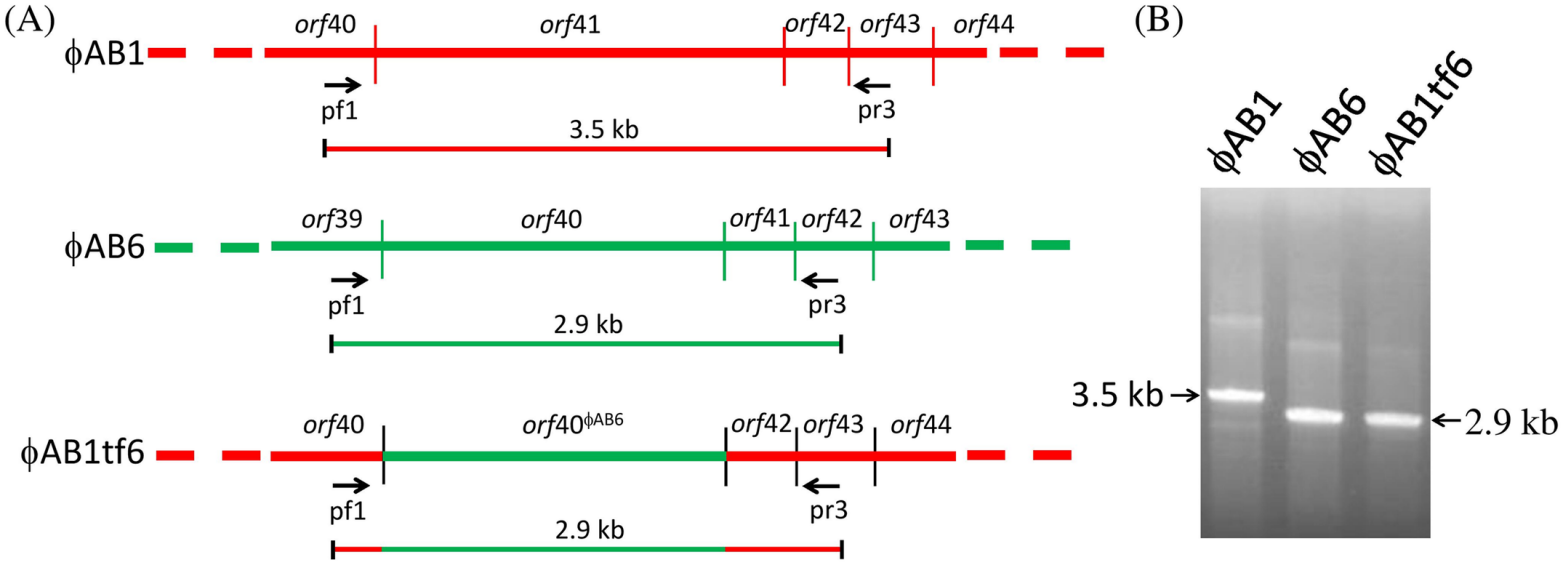
Confirmation of chimeric phage ϕAB1tf6 by PCR. (A) Maps of *orf41*^ϕAB1^/*orf40*^ϕAB6^ and the surrounding genes. (B) The PCR reactions were performed on the genomes of ϕAB1, ϕAB6, and ϕAB1tf6 as the templates with pf1 and pr3 primers and the amplicons were subjected to separation in 0.8% agarose gel.

**Fig 6:**
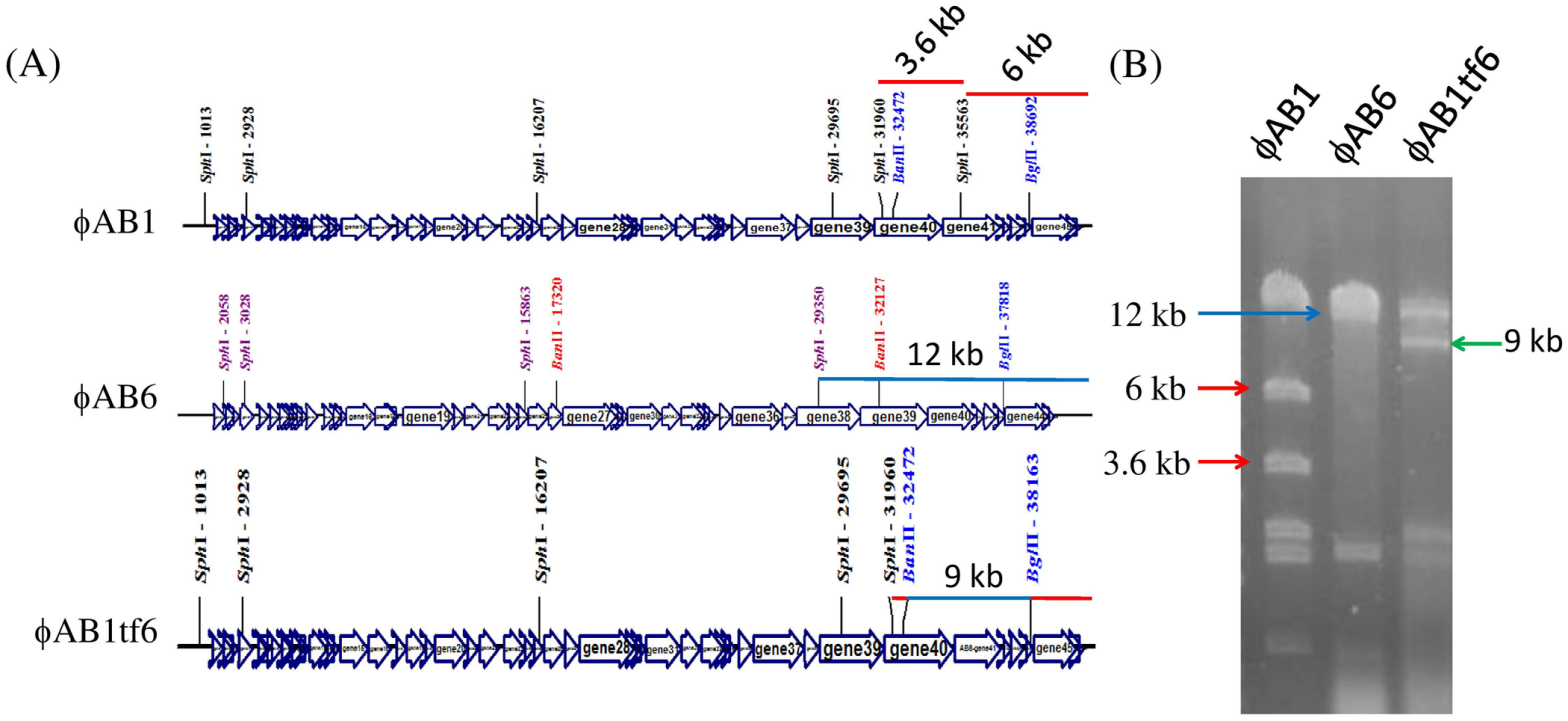
Confirmation of chimeric phage ϕAB1tf6 by RFLP. (A) The *Sph*I-restriction map shows *orf41*^ϕAB1^/*orf40*^ϕAB6^ and surrounding genes. (B) RFLP was performed on genomic DNA of ϕAB1, ϕAB6, and ϕAB1tf6 with *Sph*I digestion. The digests were subjected to separation in 0.8% agarose gel.

**Fig 7:**
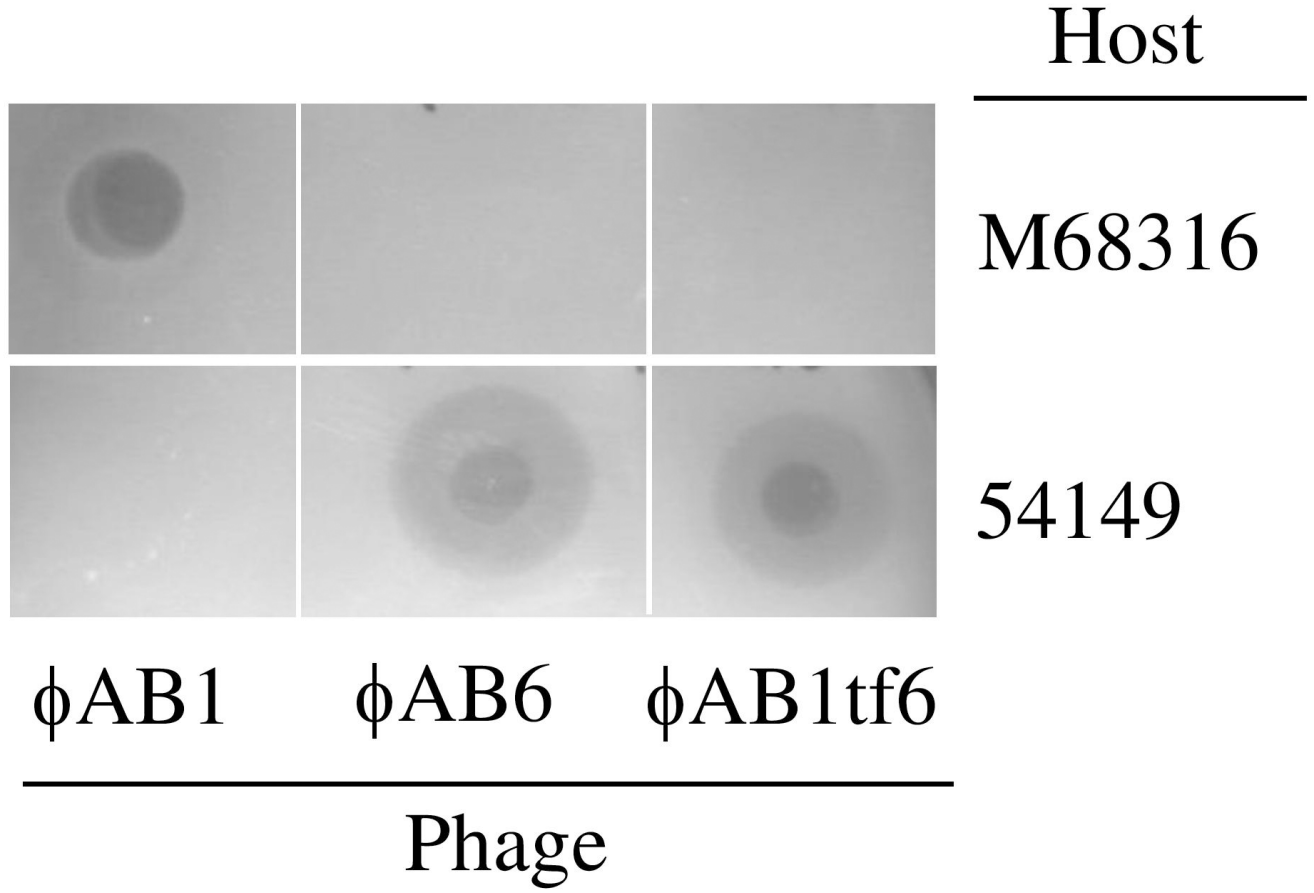
Spot assay for testing the infection ability of ϕAB1, ϕAB6, and ϕAB1tf6 for *A*. *baumannii* strains 54149 and M68316.

**Fig 8:**
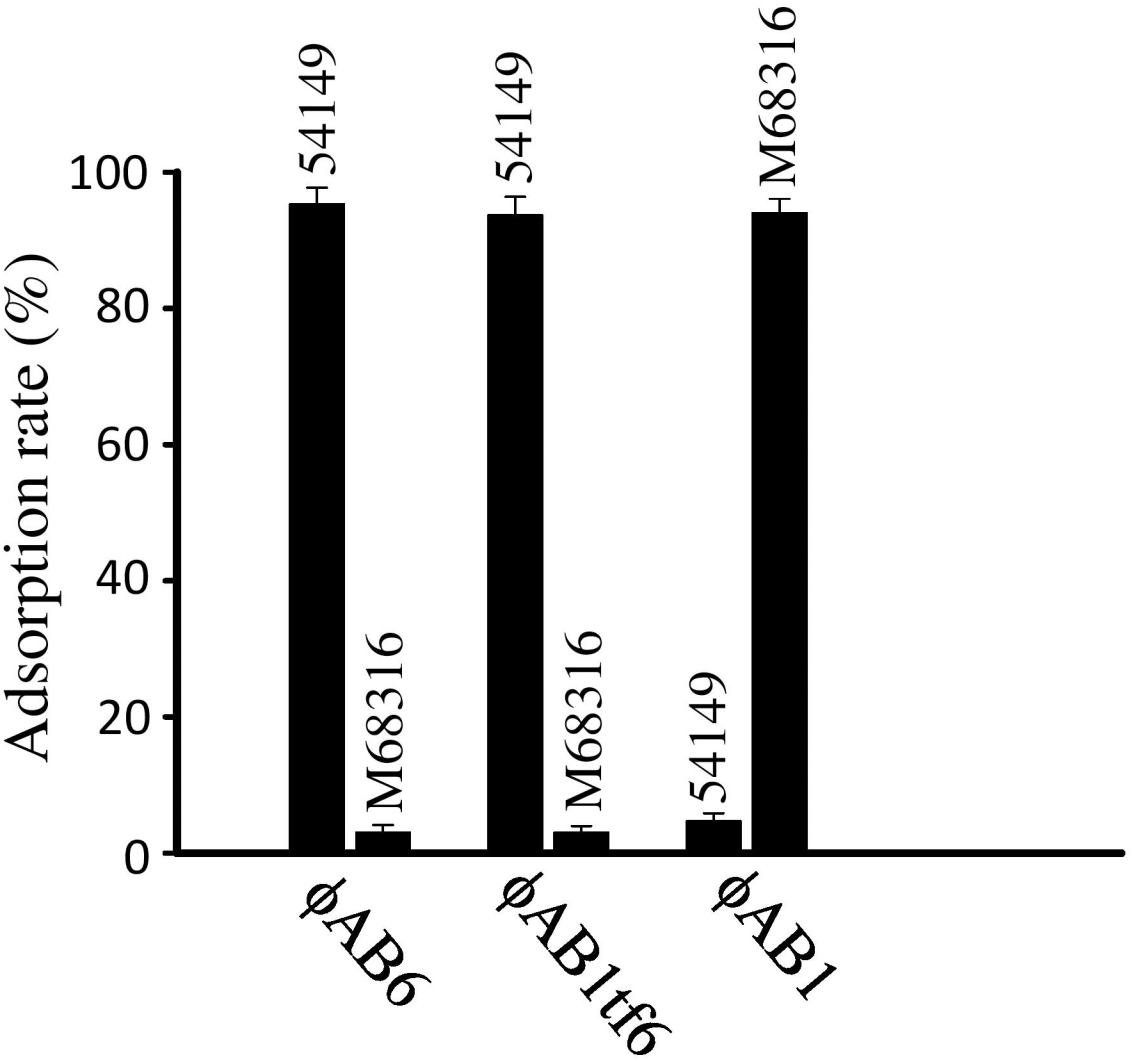
Adsorption assay for testing the specificity of ϕAB1, ϕAB6, and chimeric phage ϕAB1tf6, as described in Materials and Methods, in binding to *A*. *baumannii* strains M68316 and 54149.

Given that the phage tail fiber is often responsible for the adsorption specificity of phages [5,18], we hypothesized that replacement of ORF41 in phage ϕAB1 with ORF40 from phage ϕAB6 would lead to infection of strain 54149 instead of strain M68316. We therefore constructed the chimeric phage ϕAB1tf6. The resulting chimeric ϕAB1tf6 phage was confirmed by PCR using pf1 and pr3 primers, which were designed to amplify a 3.5-kb fragment from ϕAB1 and a 2.9-kb fragment from ϕAB6. When *orf41*^ϕAB1^ was successfully replaced by *orf*40^ϕAB6^, i.e., ϕAB1tf6 was used as the template, pf1-pr3 primers amplified a PCR product of the same size as that from ϕAB6 (Fig 5). In addition, *Sph*I-digested restriction fragment length polymorphism (RFLP) was used to verify that gene swapping had indeed occurred. As shown in Fig 6, the restriction pattern of ϕAB1tf6 (1, 2, 2.2, 9, and >12 kb) was almost identical to that of ϕAB1 (1, 2, 2.2, 3.6, 6, and >12 kb) except that the 3.6- and 6-kb fragments in ϕAB1 were missing and replaced by a 9-kb fragment, indicating that the *orf41* region was derived from ϕAB6. Both the PCR and RFLP results indicated that the obtained chimeric phage ϕAB1tf6 was recombinant and not a contamination of other unrelated phages. Spot assays showed that the chimeric ϕAB1tf6 was able to form clearing zones on strain 54149 but not on the original host strain M68316 for ϕAB1 (Fig 7). To test if the inability of ϕAB1tf6 to infect M68316 was due to a defect in phage adsorption, an adsorption assay was performed. The result (Fig 8) showed that ϕAB1tf6 displayed strong binding to 54149 but limited adsorption to M68316. The adsorption capability observed for the chimeric ϕAB1tf6 was similar to that of ϕAB6 but different from that of its parental phage ϕAB1. These data indicated that replacement of the tail fiber gene changed the host specificity of phage ϕAB1.

## Discussion

Multidrug-resistant *A*. *baumannii* causes severe nosocomial infections among immunocompromised individuals [19]. Due to the paucity in development of novel antibiotics, phage therapy has been proposed as a potential alternative treatment for multidrug-resistant *A*. *baumannii* infection. However, knowledge about the mechanisms of phage infectivity and phage resistance in *A*. *baumannii* remains limited. In particular, little is known about the initial irreversible adsorption of *A*. *baumannii* phage to its host cells, a key step in the specific interaction between host receptor and phage RBP. Phage RBPs have been proposed as potential targets for genetic modification to broaden the host range, thus contributing to practical applications of phage therapy and the detection of bacteria by their specific phages [2,4,20]. In this study, we sequenced one of the *A*. *baumannii* podophages, ϕAB6, and showed its high similarity to the previously sequenced phage ϕAB1. Despite their high sequence similarity, ϕAB1 and ϕAB6 exhibited distinct host specificity. Detailed comparative sequence analysis revealed striking sequence diversity between the C-termini of the putative tail fibers of ϕAB1 and ϕAB6. This finding is similar to previous studies showing that the RBPs of *Lactococcus lactis* phages sk1, bIL170, and TP901-1 have a conserved N-terminus and a variable C-terminus that serves as the host specificity determinant [3,21]. Similarly, in λ phage, the C-terminal domain of the tail fiber protein is responsible for binding to the receptor protein LamB [7,22]. To test whether this domain was responsible for host specificity in *A*. *baumannii* podophages, we constructed a chimeric phage ϕAB1tf6 by replacing ORF41 in phage ϕAB1 with the corresponding gene (ORF40) from ϕAB6. The resultant chimeric phage ϕAB1tf6 acquired the host range of phage ϕAB6, demonstrating that the tail fiber protein is responsible for host specificity and that tail fiber substitution in related phages can lead to acquisition of different host ranges.

Further, our spot tests revealed that ORF40^ϕAB6^ exhibited exopolysaccharide depolymerase activity toward *A*. *baumannii*, but no depolymerase activity was detected for ORF41^ϕAB1^. This result suggests that the pectate lyase 3 domain is responsible for the exopolysaccharide depolymerase activity. To our knowledge, no phage-derived enzymes that degrade the *Acinetobacter* exopolysaccharide matrix have been previously described, even though polysaccharide depolymerases are common constituents of tail spikes among phages [17] and include the activities of endorhamnosidases [23,24], alginate lyases [25], endosialidases [26], and hyaluronidases [27]. Furthermore, the pectate lyase 3 domain of ORF40^ϕAB6^ is unique among reported phage exopolysaccharide depolymerases, suggesting that it represents a novel phage-encoded exopolysaccharide depolymerase. Our results suggest that host exopolysaccharides are the specific receptor for ϕAB6, since neither the phage nor the purified ORF40^ϕAB6^ causes lysis or translucent halo formation on the host strain of ϕAB1. The specificity of ϕAB6 could thus be due to the phage-encoded polysaccharide depolymerase, which can recognize the *A*. *baumannii* 54149 exopolysaccharide structure distinct from other exopolysaccharide types. Since exopolysaccharide depolymerase is capable of destroying the outer layer of bacteria, which in turn reduces biofilm formation and antibiotic resistance by impeding drug penetration, ORF40^ϕAB6^ has the potential to be developed into a biofilm-degrading enzyme for controlling *A*. *baumannii* infections.

To our knowledge, this is the first report on the identification of a phage genetic determinant involved in *A*. *baumannii* host recognition, an RBP that possesses polysaccharide depolymerase activity. The well-characterized host specificity, the full genomic sequence of phage ϕAB6, and the polysaccharide depolymerase revealed in this study will be helpful for further elucidating the mechanistic details that determine the host range of this phage. The depolymerase activity of the ORF40^ϕAB6^ could be applied toward revealing exopolysaccharide structures. Finally, polysaccharides can be used as immunogens for glycoconjugated vaccine production [28]. Our data indicating that ϕAB6 is able to lyse 58% (483/832) of the clinical *A*. *baumannii* isolates suggest that vaccine prepared from the ORF40^ϕAB6^-digested *A*. *baumannii* polysaccharide could possibly protect from over half of the infections caused by *A*. *baumannii* in Taiwan.
